# LPCF: Robust Correlation Tracking via Locality Preserving Tracking Validation

**DOI:** 10.3390/s20236853

**Published:** 2020-11-30

**Authors:** Yixuan Zhou, Weimin Zhang, Yongliang Shi, Ziyu Wang, Fangxing Li, Qiang Huang

**Affiliations:** 1 School of Mechatronical Engineering, Beijing Institute of Technology, Beijing 100081, China; 3120180180@bit.edu.cn (Y.Z.); ylshi@bit.edu.cn (Y.S.); 3120180174@bit.edu.cn (Z.W.); wonk2000@bit.edu.cn (F.L.); qhuang@bit.edu.cn (Q.H.); 2 Key Laboratory of Biomimetic Robots and Systems, Beijing Institute of Technology, Ministry of Education, Beijing 100081, China; 3 Beijing Advanced Innovation Center for Intelligent Robots and Systems, Beijing 100081, China

**Keywords:** object tracking, correlation filter, decontamination, model drift, locality preserving

## Abstract

In visual tracking, the tracking model must be updated online, which often leads to undesired inclusion of corrupted training samples, and hence inducing tracking failure. We present a locality preserving correlation filter (LPCF) integrating a novel and generic decontamination approach, which mitigates the model drift problem. Our decontamination approach maintains the local neighborhood feature points structures of the bounding box center. This proposed tracking-result validation approach models not only the spatial neighborhood relationship but also the topological structures of the bounding box center. Additionally, a closed-form solution to our approach is derived, which makes the tracking-result validation process could be accomplished in only milliseconds. Moreover, a dimensionality reduction strategy is introduced to improve the real-time performance of our translation estimation component. Comprehensive experiments are performed on OTB-2015, LASOT, TrackingNet. The experimental results show that our decontamination approach remarkably improves the overall performance by 6.2%, 12.6%, and 3%, meanwhile, our complete algorithm improves the baseline by 27.8%, 34.8%, and 15%. Finally, our tracker achieves the best performance among most existing decontamination trackers under the real-time requirement.

## 1. Introduction

Visual tracking, in general, refers to the task of estimating locations and sizes of an arbitrary target in image sequences with only its initial states. Although great progress [1,2,3,4,5,6,7,8,9] has been acquired in this field over the past decades, yet it remains to be particularly challenging due to partial or complete occlusions, severe variations in scale, cluttered backgrounds, complex object motion, and real-time processing requirements.

Recently, deep learning-based methods [7,8,9,10,11] have dominated this filed and achieved very promising performances, as well as very fast speed (e.g., DaSiamRPN [12] 160FPS). Nevertheless, most deep learning-based methods rely on training on expensive GPUs with gigantic quantities of data. Therefore, it is still challenging and meaningful to explore efficient non-deep-learning methods. Among most non-deep-learning methods, there exist two main methods to deal with visual tracking, namely generative and discriminative methods. Generative trackers [13,14,15] handled the problem by finding the most matched regions of the target model. Templates are mostly utilized in those methods. Discriminative approaches [2,3,16] took tracking as differentiating the object from the background, which refers to a classification problem. Thanks to the development of several large benchmark datasets, especially OTB-2015 [17], TrackingNet [18], and LASOT [19], the research on this specific computer vision problem has gained great progress. Over those improvements, discriminative approaches, particularly the correlation filter (CF) based trackers have exhibited very promising performance with high computational efficiency.

The main inspiration of the correlation filter conceives in the Convolution Theorem, proving the fact that the correlation computation in the time domain is equivalent to an element-wise multiplication in the Fourier domain. The correlation filter was first introduced into visual tracking in 2010 by Bolme, who proposed learning a Minimum Output Sum of Squared Error (MOSSE [1]) filter updated frame-by-frame to represent the objective appearance and accomplish the tracking task on gray images. MOSSE profited from the superior computational efficiency of the correlation filter to achieve a speed of hundreds of frames per second. Furthermore, Henriques et al. [2] explored a dense sampling strategy by approximating the sample window displacement as cyclic shifts, effectively promoting the tracker’s discriminative power. This strategy, along with the Fast Fourier Transform constitutes the foundation of CF-based trackers. Thereafter, powerful handcrafted features like Histogram of Oriented Gradient (HOG [3]) and deep features [8] were integrated into the correlation filter framework and further promoted its performance. Moreover, Danelljan et al. [20] proved that the correlation filter also performs superior in addressing the scale variations besides translation estimation.

To adjust to unpredictable variations in the appearance of the objective during the tracking process with little prior knowledge, both generative and discriminative trackers must update their model online, so does the CF-based trackers. However, when encountering with blurred appearance, inaccurate predictions, and out-of-plane rotation that cause training samples to be in misalignment, most trackers [8,15,21] ignored this problem and updated their model continually. As a consequence, the model drifted, eventually inducing tracking failure. Also, occlusions containing background information, were indiscriminately updated as positive training samples and thus deteriorated the discriminative power of the model. In visual tracking, this problem is the so-called training set decontamination [22] or model drift.

Recently, although trackers generally neglected the decontamination problem, several works had investigated the model drift problem in the model update phase. Bolme et al. [1] developed MOSSE to determine whether new samples should be rejected or not based on the Peak-to-Sidelobe Ratio (PSR) criterion. Kala et al. proposed PN [23] and defined tracking confidence as Normalized Cross Correlation (NCC) between the tracked patch and the initial patch. Zhong et al. proposed SCM [24] and announced an invalid tracking result if the occlusion condition of the tracking result in the new frame was bigger than a pre-defined one. Kala et al. proposed TLD [25] and declared a failure of the tracker if the median distance was larger than a pre-defined threshold. In similar, Supancic et al. [26] used the formalism of self-paced curriculum learning to automatically select the correct frames and retraining the model with these frames. Tu et al. [13] took both reconstruction confidence and occlusion into account and proposed a multi-memory weight allocation strategy for template updating. Zhang et al. proposed the Multi-Expert Entropy Minimization (MEEM [27]) algorithm combining multiple experts (a tracker and its historical snapshots) and activating the restore scheme based on a designed entropy criterion. Gao et al. tackled the problem by considering distances between all pairs of samples. Ma et al. [28] used the naive correlation response maximum to activate the re-detection scheme, where they trained a random fern classifier to re-detect objects. Wang et al. [29] proposed a novel criterion called average peak-to-correlation energy (APCE) and rejected deteriorated samples if the APCE and the maximum response of the current frame are both larger than their corresponding historical mean with pre-defined ratios. The APCE represents the fluctuated level of correlation response maps. Similar to our method, Hong et al. proposed Muster [30] and developed a keypoint-based method maintaining a database to ascertain occlusions. Different from trackers adopting an explicit training sample management component, Danelljan et al. proposed a unified formulation by minimizing a single loss over both the target appearance and the sample quality weights to down weight corrupted samples [22].

In summary, Since CF-based trackers all calculate the correlation response map between two consecutive frames, it is intuitionistic to utilize this nature. Therefore these CF-based trackers either employed the naive correlation response maximum [28] alone, a custom-designed criterion using the response [1] or a combination [29]. Other different approaches either took advantage of a combination of experts [23,27], or validated the tracking results under physic constraints [24,25,30].

In this work, we investigate the problem mentioned above and propose a real-time scale-adaptive and robust tracker called locality preserving correlation filter (LPCF) to tackle this problem. Similar to the work in Reference [30], our tracking results validation algorithm also based on feature points. Nevertheless, our approach is different from the Muster [30], which only took the number of matched features points between the current frame and maintained database as a tracking failure clue. We preserve the locality containing spatial neighborhood relationships and topological structures of the tracking bounding box center. A closed-form solution of our locality preserving approach is derived and discussed in detail in Section 3.

The main contributions of this work could be concluded as follows.

- We extend the translation estimation component with a generic scale estimation approach, which has shown to obtain excellent performance for addressing visual tracking scale variation.
- We resample the feature in different sizes. Furthermore, PCA is introduced to reduce the computational cost of the translation estimation approach and to antagonize minor disturbance. This PCA method increases the real-time performance of the translation estimation approach without sacrificing its robustness.
- We derive a linearithmic complexity solution of locality-preserving tracking validation and adjust it for the practical tracking process.
- Extensive experiments on challenging large datasets OTB-2015, TrackingNet, and LASOT are performed. The results demonstrate that the presented decontamination method is effective and it increases the baseline remarkably in the AUC score on all three datasets. What’s more, the experimental results also show that the complete tracker performs approvingly against other decontamination trackers and state-of-the-art methods.

## 2. Materials and Methods

**Figure 1:**
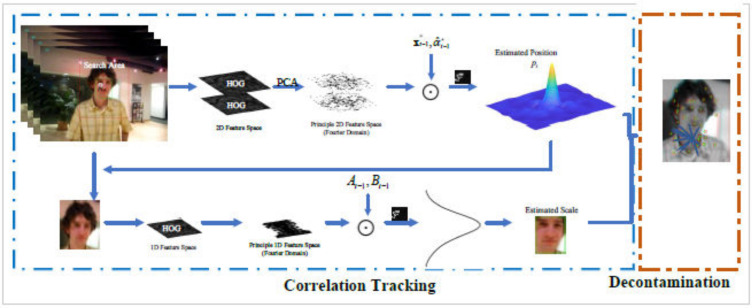
Overview of the proposed algorithm The tracking task is decomposed into three parts, namely translation, scale estimation, and decontamination. The position $p_{t}$ is infered from the correlation response map using translation model ${\hat{x}}_{t-1}^{⋆},{\hat{\alpha }}_{t-1}^{⋆}$, and the scale $s_{t}$ is predicted using scale model $A_{t-1},B_{t-1}$. After both position and scale are estimated, the proposed keypoint-based decontamination approach is used to choose the reliable frame to update model.

In this section, our algorithm is described in detail. Firstly, we describe the kernelized correlation filter (KCF [3]). After that, we discuss the dimensionality reduction strategy to decrease the computational cost of standard KCF. Finally, we present an online tracking result validation scheme to accomplish the decontamination task, as well as to prevent model drift. Figure 1 shows an overview flowchart of our algorithm.

### 2.1. Revisist of Kernelized Correlation Filter

Generally, the kernelized correlation filter trains a classifier from the initial frame using a single sample z of the target appearance. Here, the sample z corresponds to features extracted from a rectangular image patch centered around the target. In order to generate dense samples from a single sample z, cyclic shifts are employed to approximate the search window displacement. Due to the cyclic property, the patch wraps around on boundary positions, causing some distortion compared to the true displacement. However, appropriate padding and windowing can mitigate this undesirable property [3]. Thus the sample patch size is usually twice larger than the target size. The objective of correlation filter is to learn w, attained by minimizing the L2 errors of the correlation response over the expectable same size Gaussian function label y,

(1) $$\min_{w}∥w^{T}{z-y∥}_{2}^{2}+\lambda {∥w∥}^{2},$$

where the non-negative constant λ is the regularization parameter. Using “kernel trick” allows more powerful, non-linear regression function. Here KCF [3] uses Gaussian kernel. According to Representer Theorem, the alternative representation α is said to be in the dual space, as opposed to the primal space w, Equation (1) can be written as

(2) $$\min_{\alpha }{∥\sum_{i=1}^{n}\alpha_{i}k\left(z,x_{i}\right)-y∥}_{2}^{2}+\lambda \alpha^{T}K\alpha ,$$

where $k(z,x_{i})$ is the kernel function of testing sample z between all training examples $x_{i}$ and K is the positive semi-definite kernel matrix with $K_{ij}=k\left(x_{i},x_{j}\right)$ as its elements.

Equation (2) can be solved efficiently using Discrete Fourier Transform (DFT) to transfer into the Fourier domain. The closed-form desired filter α is given by

(3) $$\hat{\alpha }=\frac{\hat{y}}{{\hat{k}}^{xx}+\lambda },$$

where the hat denotes the DFT result, $k^{xx}$ denotes kernel function of training examples x between itself. In the tracking step, the position estimation is achieved on the same size sample z in a fresh frame by calculating the response map as

(4) $$R=F^{-1}\left({\hat{k}}^{x^{⋆}z}⊙{\hat{\alpha }}^{⋆}\right),$$

where ⊙ denotes the elementwise product and $x^{⋆},\alpha^{⋆}$ is the learned target model. The translation vector from last frame to current frame is then estimated using the index of the maximum value of response scores. The model is updated by linear combination in the Fourier domain

(5) $$\begin{array}{c} {{\hat{x}}^{⋆}}_{t}=\left(1-\delta \right){{\hat{x}}^{⋆}}_{t-1}+\delta {\hat{x}}_{t}\\ {{\hat{\alpha }}^{⋆}}_{t}=\left(1-\delta \right){{\hat{\alpha }}^{⋆}}_{t-1}+\delta {\hat{\alpha }}_{t}. \end{array}$$

### 2.2. Proposed Approaches

#### 2.2.1. Dimensionality Reduction Strategy

Standard KCF only estimated the displacement of the target between two consecutive frames. However, scale variations are such a common circumstance over visual tracking. We incorporate scale estimation with standard KCF by adopting a scale search strategy proposed by Reference [20]. However, the scale-estimation extension although handles scale variations robustly but still sacrifices some real-time performances. Thereafter, inspired by the work of Reference [20], we extend the same sub-grid responses interpolation and feature dimensionality reduction scheme for standard KCF.

According to Danejjan [20], the computational cost of the CF-based trackers is determined by the number of FFT computations. Thus, to reduce the required number of FFT operations, we extend standard KCF with the standard PCA. Similar to Reference [20], we update the target template using $u_{t}=\left(1-\delta \right)u_{t-1}+\delta x_{t}$, then use this compressed sample to generate the projection matrix $P_{t}$ by minimizing the reconstruction error of the target template $u_{t}$.

(6) $$\min_{P_{t}}\sum_{n}{∥u_{t}\left(n\right)-P_{t}^{T}P_{t}u_{t}\left(n\right)∥}^{2}.$$

Then the filter is updated as

(7) $${\hat{x}}_{t}^{⋆}=F\left\{P_{t}x_{t}\right\},$$

where F denotes the FFT operation. In our kernelized case, we update the ${\alpha^{⋆}}^{t}$ using (4) and (5).

#### 2.2.2. Decontamination

The absolute Euclidean distance between two feature points extracted from the same target can vary significantly under viewpoint changes or non-rigid deformations in two adjacent frames of one tracking target. Nevertheless, according to the consecutive nature (only restricted change occurred between continuous frames) of visual tracking, the spatial neighborhood relationship among feature points is generally well preserved due to physical constraints. The right bounding box center represents the physic center of the tracking target, which naturally keeps consistent in two adjacent frames. Therefore, we consider that the pair of putative center correspondence between two consecutive frames is also a ‘virtual’ feature pair.

Since we consider the pair of centers is also a ‘virtual’ feature pair, inspired by the LPM [31], we can verify whether the centers pair is an outlier or not. Considering $I=\{x_{i},y_{i}\}$, $C=\{c_{1},c_{2}\}$, S={I,C} where $c_{1},c_{2}$ and $x_{i},y_{i}$ are 2D vectors containing coordinates of centers and feature points. Because the spatial neighborhood relationship is preserved, so if the C is a unknown pair, and the I is the correct feature correspondences. The optimal solution is

(8) $$I^{*},C^{*}=\arg\min_{S}C\left(I;C;S,\eta \right),$$

with the cost function *C* defined as

(9) $$\begin{array}{c} C\left(I;C;S,\eta \right)=\frac{1}{2K}\left(\sum_{i|x_{i}\in N_{C_{1}}}{\left(d\left(c_{1},x_{i}\right)-d\left(c_{2},y_{i}\right)\right)}^{2}\right.\left.+\sum_{i|y_{i}\in N_{c_{2}}}{\left(d\left(c_{1},x_{j}\right)-d\left(c_{2},y_{j}\right)\right)}^{2}\right.\\ \left.+\sum_{i\in I}(\sum_{j|x_{j}\in N_{x_{i}}}{\left(d\left(x_{i},x_{j}\right)-d\left(y_{i},y_{j}\right)\right)}^{2}\right.\left.+\sum_{j|y_{j}\in N_{y_{i}}}{\left(d\left(x_{i},x_{j}\right)-d\left(y_{i},y_{j}\right)\right)}^{2})\right)+\eta \left(N-|I|-|C|\right), \end{array}$$

where $N_{x},N_{y}$ denotes the neighborhood contains K nearest Euclidean neighbors of point x, y, respectively. The first term uses 1/2K to normalize the contribution of each element in the neighborhood, and the second term discourages the outliers. Then use the positive coefficient η to balance the two terms.

When we consider the I is all inlier sets, which means we suppose the feature points extracted from two frames matched perfectly. Thanks to the outstanding work of RANSAC [32], it is easy to get a refined feature correspondence set I. Therefore (9) can be simplified as

(10) $$\begin{array}{c} C\left(I;C;S,\eta \right)=\frac{1}{2K}\left(\sum_{i|x_{i}\in N_{C_{1}}}{\left(d\left(c_{1},x_{i}\right)-d\left(c_{2},y_{i}\right)\right)}^{2}\right.\left.+\sum_{i|y_{i}\in N_{c_{2}}}{\left(d\left(c_{1},x_{i}\right)-d\left(c_{2},y_{i}\right)\right)}^{2}\right)+\eta \left(1-|C|\right). \end{array}$$

We associate the putative centers pair with a label p, where p ∈{0,1} represents tracking correctness. Inspired by the LPM [31], we also make a binarization operation as:

(11) $$d\left(c_{1},x_{i}\right)=\left\{\begin{array}{c} 0,x_{i}\in N_{c_{1}}\\ 1,x_{i}\notin N_{c_{1}} \end{array},\;d\left(c_{2},y_{i}\right)=\left\{\begin{array}{c} 0,y_{i}\in N_{c_{2}}\\ 1,y_{i}\notin N_{c_{2}}. \end{array}\right.\right.$$

So with this definition in (11), we consider

(12) $$\begin{array}{cc} \sum_{i|x_{i}\in N_{C_{1}}}{\left(d\left(c_{1},x_{i}\right)-d\left(c_{2},y_{i}\right)\right)}^{2} & =\sum_{i|x_{i}\in N_{C_{1}},y_{i}\in N_{C_{2}}}d{\left(c_{2},y_{i}\right)}^{2}+\sum_{i|x_{i}\in N_{C_{1}},y_{i}\notin N_{C_{2}}}d{\left(c_{2},y_{i}\right)}^{2}\\ \; & =0+\mathrm{number}(i|x_{i}\in N_{C_{1}},y_{i}\notin N_{C_{2}})\\ \; & =K-\mathrm{number}(i|x_{i}\in N_{C_{1}},y_{i}\in N_{C_{2}})\\ \; & =K-n_{i}=\sum_{i|y_{i}\in N_{C_{2}}}d{\left(c_{1},x_{i}\right)}^{2} \end{array}$$

where number(.) indicates element numbers, and $n_{i}$ refers to the number of same elements in two neighborhoods $N_{c_{1}}$ and $N_{c_{2}}$.

So the cost function in (10) can be simplfied as follows using (12).

(13) $$C\left(p;S,\eta \right)=\frac{p}{K}\sum_{i|x_{i}\in N_{C_{1}}}d{\left(c_{2},y_{i}\right)}^{2}+\eta \left(1-p\right).$$

The cost function derived above only models the distance yet ignores the topological structure. So we design another cost term to ulteriorly utilize the consistency of the neighborhood topology. Because the putative center correspondence $c_{1},c_{2}$ is a virtual feature match and thus does not share the same motion with other true feature matches, especially in a rotation case. So when encountering with tracking failure validation, we model the relative vectors $v_{i}$, between the center c and the feature points $f_{i}$ rather than the corresponding displacement vector between a putative match ($x_{i},y_{i}$) employed by the LPM.

Let $v_{1i},v_{2i}$ denote the relative vectors of $c_{1},x_{i}$ and $c_{2},y_{i}$ respectively. We specify the consistency of neighborhood topology using the length ratio and the angle between $v_{1i}$ and $v_{2i}$.

(14) $$s\left(v_{1i},v_{2i}\right)=\frac{\min\left\{d\left(c_{1},x_{i}\right),d\left(c_{2},y_{i}\right)\right\}}{\max\left\{d\left(c_{1},x_{i}\right),d\left(c_{2},y_{i}\right)\right\}}\cdot \frac{\left(v_{1i},v_{2i}\right)}{d\left(c_{1},x_{i}\right)\cdot d\left(c_{2},y_{i}\right)},$$

also we quantize the distance into two levels to adapt to the scale variation and orientation.

(15) $$d\left(v_{1i},v_{2i}\right)=\left\{\begin{array}{c} 0,s\left(v_{1i},v_{2i}\right)\geq \tau \\ 1,s\left(v_{1i},v_{2i}\right)<\tau . \end{array}\right.$$

With the above definition, the cost function in (13) can be renew as:

(16) $$\begin{array}{c} C\left(p;S,\eta ,\tau \right)=\frac{p}{K}\left(\sum_{i|x_{i}\in N_{C_{1}}}d{\left(c_{2},y_{i}\right)}^{2}\right.\left.+\sum_{i|x_{i}\in N_{C_{1}},y_{i}\in N_{C_{2}}}d\left(v_{1i},v_{2i}\right)\right)+\eta \left(1-p\right). \end{array}$$

Using a fixed *K* is not a solution for the general tracking failure detection problem. So we obtain a set of neighbors size $K={\left\{K_{m}\right\}}_{m=1}^{M}$. And the (16) becomes

(17) $$\begin{array}{c} C\left(p;S,\eta ,\tau \right)=\frac{p}{M}\sum_{m=1}^{M}\frac{1}{K_{m}}\left(\sum_{i|x_{i}\in N_{C_{1}}}d{\left(c_{2},y_{i}\right)}^{2}\right.\left.+\sum_{i|x_{i}\in N_{C_{1}},y_{i}\in N_{C_{2}}}d\left(v_{1i},v_{2i}\right)\right)+\eta \left(1-p\right), \end{array}$$

where 1/M normalizes the effort of each level of the neighborhood. To optimize the final objective function (17), we replace the complex form derivation with a simple form and obtain:

(18) $$\begin{array}{c} c=\sum_{m=1}^{M}\frac{1}{MK_{m}}\left(\sum_{i|x_{i}\in N_{C_{1}}}d{\left(c_{2},y_{i}\right)}^{2}\right.\left.+\sum_{i|x_{i}\in N_{C_{1}},y_{i}\in N_{C_{2}}}d\left(v_{1i},v_{2i}\right)\right) \end{array}$$

and therefore, the final objective function should be:

(19) C(p;S,λ,τ)=p(c−λ)+η.

The cost value c can be calculated in advance, and hence the solution of this objective function is

(20) $$p=\left\{\begin{array}{c} 1,\;c\leq \eta \\ 0,\;c>\eta \end{array}\right.$$

and so far, the tracking failure is determined by the *p* value.

In real tracking practice, we evaluate the confidence $c_{a},c_{m}$ of $f_{t-5}$, $f_{t-1}$ between $f_{t}$ respectively. Then fuse them using $c=\beta c_{a}+\left(1-\beta \right)c_{m}$ where β denotes weight. Thus the tracking failure is determined by the c>β. The proposed method overview is summarized in Algorithm 1.
**Algorithm 1** Proposed tracking algorithm**Input** Image $f_{t}$, Previous target state ($p_{t-1}$, $s_{t-1}$). Translation Model ${\hat{x}}_{t-1}^{⋆},{\hat{\alpha }}_{t-1}^{⋆}$, Scale Model $\;A_{t-1},B_{t-1}$.**Output** Estimated state ($p_{t}$, $s_{t}$), Translation Model ${\hat{x}}_{t}^{⋆},{\hat{\alpha }}_{t}^{⋆}$, Scale Model $\;A_{t},B_{t}$. **repeat**     *// Translation estimation*     Extract sample $z_{t}$ from $f_{t}$ at $p_{t-1}$ and $s_{t-1}$.     Do feature dimensionality reduction.     Compute response map $R_{t}$ and estimate $p_{t}$ using (4).     *// Scale estimation*     Estimate $s_{t}$ according to Reference [20].     *// Decontamination*     Extract SURF feature points set $S_{t}$ from $f_{t}$ at $p_{t}$ and $s_{t}$.     Compute the tracking derivation *c* using $S_{t},S_{t-1},S_{t-5}$.     *// Model update*     Extract sample $z_{t}$ from $f_{t}$ at $p_{t}$ and $s_{t}$.     Do feature dimensionality reduction.     **if** c>T **then**          Update the translation model ${\hat{x}}_{t}^{⋆},\;{\hat{\alpha }}_{t}^{⋆}$ using (5)–(7).     **end if**     Update the scale model $\;A_{t},B_{t}$ [20]**until** (the sequence ends)

## 3. Results and Discussion

### 3.1. Implemental Details

We implement the developed algorithm by primal MATLAB without any optimization. All the experiments are conducted on an Intel I7-7700HQ CPU@2.89GHZ with 16GB RAM. The learning rate δ of the update scale model and appearance model is set to 0.01. The padding, Gaussian variance σ and regularization parameter λ is set to 2, 0.1 and 0.01 respectively. The parameter τ,η,K,β used in tracking validation is set as 0.9/0.8, 0.8, [4, 6, 8], 0.6 respectively. Similar to Fast Discriminative Scale Space Tracker (FDSST [20]), 33 number of scales with a scale factor of 1.02 is used in the scale model. The HOG cell size is 4×4, and the orientation bin number of HOG is 9.

For OTB-2015 results, We report our approach performance under the OTB-2015 protocol with OPE (one-pass evaluation), TRE (temporal robustness evaluation), and SRE (spatial robustness evaluation). For further information, we recommend the paper of OTB-2015. We also report our approach performance under the LASOT and TrackingNet protocol.

### 3.2. Baseline Experiments

**Figure 2:**
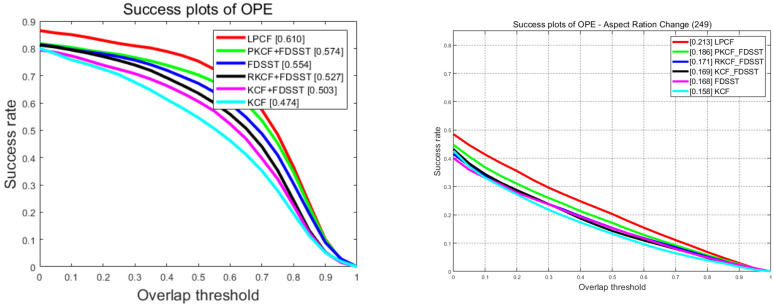
The success plots of OPE on OTB-2015 (**Left**) and LASOT (**Right**). The numbers in the legend indicate the area-under-the-curve (AUC) score. The code of KCF and FDSST is provided by the original authors. The LASOT pre-computed results of KCF and FDSST are provided by the LASOT authors. We recommend checking this figure in color.

In this work, we accomplish the simple integration of standard KCF and FDSST at first. Then we investigate the feature dimensionality reduction strategy using Principle Component Analysis (PCA). Additionally, we further extend the tracker with the proposed locality preserving the tracking validation component. We name the three stages of our tracker as KCF+FDSST, PKCF+FDSST, and LPCF, respectively. Then we report the results on OTB-2015 (100 sequences) and LASOT (280 sequences), using the area-under-the-curve (AUC) denoting the overall performance in Figure 2. For more detail analysis, we also report the tracking performance on OTB-2015 using frame-per-second (FPS), distance precision at 20 pixels and mean overlap precision computed as the percentage of frames where the intersection-over-union (IOU) overlap with the ground-truth exceeds a threshold of 0.5, in Table 1.

Table 1 shows that although the simple integration of standard KCF and FDSST improves performance in the OP term with a gain of 3.9%, compared to standard KCF, but it sacrifices precision and real-time performance with a decrease of 1.9% and 66%. KCF only uses the initial padded size to extract features, hence the naive incorporation of a scale estimation component will not achieve competitive performances. Therefore, we implement a resize KCF with FDSST, named RKCF+FDDST, which resizes the image patch of the newly estimated size into the initial size. And this strategy is inspired by the work of SAMF [33].

Figure 2 shows that the resize strategy improves the KCF+FDSST significantly with a gain of 3% and 4.3% in the OP and DP terms, while improves the overall performance with a gain of 2.4% on OTB-2015 and 0.2% on LASOT. However, the resize strategy further increases the computational burden leading to a 20% decrease of FPS. And it inevitably induces distortion of images for resizing all of them into the same initial size, especially when encountering large scale variations. Therefore, we investigate the PCA approach not only to enhance the computational efficiency but also to extract the principal component to mitigate the distortion problem. The figure proves that this strategy is effective and efficient. Besides, the strategy achieves a gain of 7.1%, 1.7% against KCF+FDSST and 4.7%, 1.5% against RKCF+FDSST in the AUC score on two datasets. What’s more, our final tracker (LPCF) equipped with training sets management component, achieves an overall performance gain of 3.6% and 2.7%, contrasted to the baseline tracker without this component. Benefiting from our well-established closed-form derivation along with the PCA approach mentioned above, our tracker still achieves real-time (30.63 FPS), while still suffers from the high computational burden of feature extraction.

TrackingNet [18] contains 511 videos collected from the YouTube website. The evaluation results of our baseline trackers are shown in Table 2. Our approach remarkably improves the AUC score (success in Table 2) with a gain of 7.2% against KCF, which demonstrates the generalization ability and effectiveness of our approach.

### 3.3. Comparison with Other Decontamination Trackers

We performed a comprehensive comparison with 8 decontamination trackers: MOSSE [1], SCM [24], TLD [25], MEEM [27], LMCF [29], LCT [28], Muster [30], SRDCFDecon [22], LCT+ [34]. Other three trackers PN [23], TCMA [13] and SPLTT [26] mentioned above are not accessible to their source code or OTB-2015 results, so we do not present them in this comparison. Although Ma et al. further extended LCT [28] into LCT+ [34] by integrating more features but keep its re-detection scheme, so we also compared our approach with it.

**Figure 3:**
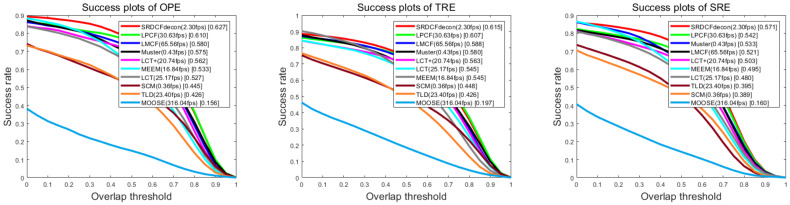
The success plots of OPE, TRE, SRE on OTB-2015. Decontamination trackers are presented at this figure. The numbers in the legend denotes the average AUC scores for success plots. The FPS is also shown in the legend.

A comparison with decontamination trackers on the OTB-2015 is shown in Figure 3. For details, we recommend checking the figure in color. Among the compared tracking algorithms, SRDCFDecon, LMCF, and Muster provide the best results with AUC scores of 62.7%, 58.0%, and 57.5%, respectively. Our algorithm performs the second results with an AUC score of 61.0%. Nevertheless, our method performs superior in speed and can run at 30.63 FPS, which meets the real-time requirements, whereas SRDCFdecon can only run at 2.7 FPS. And our training sets management method significantly improves our baseline tracker with an increase of 3.6% AUC score, yet SRDCFdecon only improves its baseline tracker with an increment in 1.1% AUC score.

To demonstrate the robustness of our tracker, we follow the protocol of OTB-2015. The robustness is evaluated using SRE and TRE. The first criterion SRE shifts the bounding boxes to initialize the tracker at different locations. TRE splits a video into several fragments to initialize the tracker at different frames. We present the success plots for SRE and TRE in Figure 3. In both cases, our algorithm achieves robustness. It leads to a coincident performance promotion in both cases.

#### 3.3.1. Comparison with State-of-the-Art Trackers

**Figure 4:**
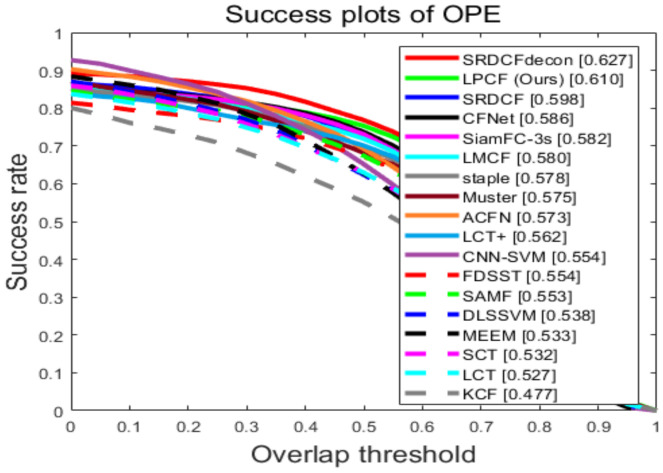
The success plots of OPE on OTB-2015. The numbers in the legend indicate the AUC scores. Our approach LPCF (marked as green square) shows its competitive performances against other state-of-the-art trackers with 61.0% score.

**Figure 5:**
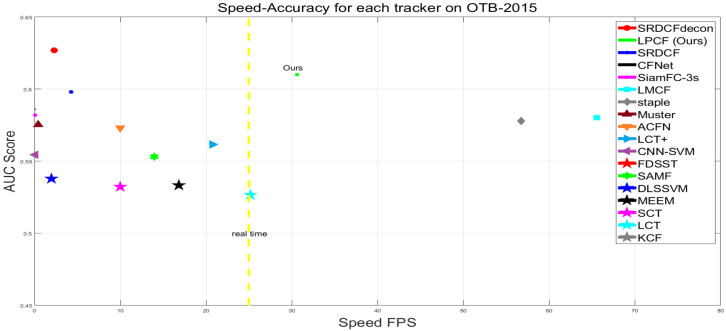
Speed and accuracy plot of state-of-the-art visual trackers on OTB-2015. The proposed LPCF algorithm achieves the best accuracy among all real-time (faster than 25 FPS) trackers.

Results on OTB-2015: For further illustrate performances of our tracker, we compare our tracker to 17 SOTA trackers on OTB-2015: LCT+ [34], LMCF [29], KCF [3], FDSST [20], SRDCF [5], staple [21], LCT [28], Muster [30], MEEM [27], CNN-SVM [35], DLSSVM [36], SCT [37], SAMF [33], CFNet [8], SiamFC-3s [6], ACFN [38]. Among these trackers, CNN-SVM, CFNET, SiamFC-3s, ACFN are deep-learning-based trackers. The comparison results are shown in Figure 4. Furthermore, a speed-accuracy plot (Figure 5) is also presented for further illustrate performances of our tracker.

Figure 4 and Figure 5 demonstrate the performance of LPCF with 17 SOTA trackers. Although presented LPCF scores the second following the SRDCFDecon tracker on the AUC score, the tracking speed is ten times faster than SRDCFDecon from its reported results at about 2.4 FPS, which severely restricts its application. Muster is the most related approach to LPCF due to a similar keypoints-based method. But LPCF performs superior of it on both overall and real-time performance because of our effective method of evaluating tracking failure and mitigating the distortion of resizing. Moreover, LPCF only utilizes a handcrafted feature HOG, whereas still performs favorably in both evaluations against SAMF, LCT+, and SRDCF fusing several features. Additionally, CFNet, SiamFC-3s, and ACFN exploiting deep features or designing deep structure also cannot compete with our tracker.

**Figure 6:**
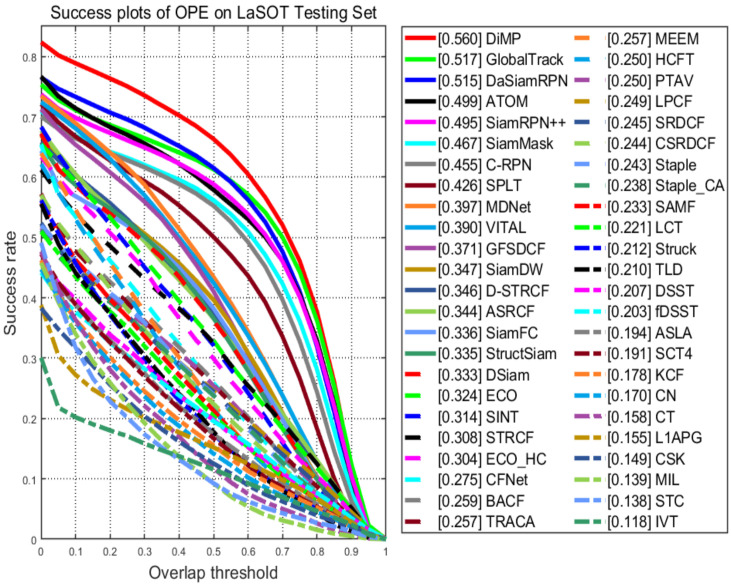
The success plots of OPE on LASOT. The numbers in the legend indicate the AUC scores. Our approach LPCF achieves 24.9% in terms of AUC score.

**Figure 7:**
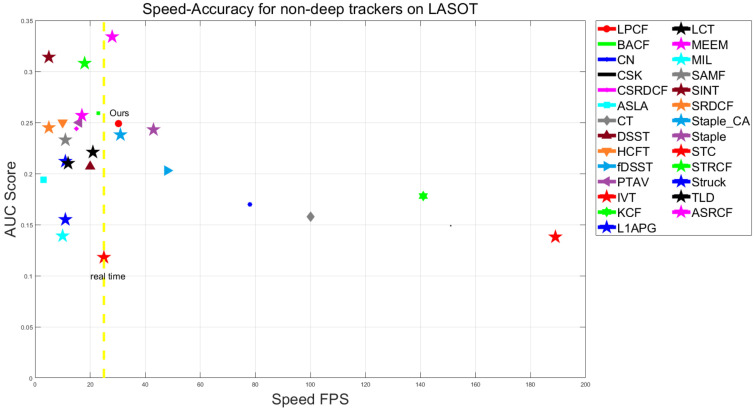
Comparison with 26 non-deep-learning trackers. Our proposed tracker LPCF (marked as red circle) achieves the second accuracy among all real-time (faster than 25 FPS) trackers.

Results on LASOT: We also compare our tracker to 47 SOTA trackers on LASOT. The 47 SOTA trackers are DiMP [10], GlobalTrack [39], DasiamRPN [40], ATOM [9], SiamRPN++ [41], SiamMask [42], C-RPN [43], SPLT [44], MDNet [45], VITAL [46], GFSDCF [11], SiamDW [47], D-STRCF [48], STRCF [48], ASRCF [49], SiamFC [6], StructSiam [50], DSiam [12], ECO [7], ECO_HC [7], CFNet [8], BACF [51], TRACA [52], MEEM [27], HCFT [53], PTAV [54], SRDCF [5], CSRDCF [55], Staple [21], Staple_CA [56], SAMF [33], LCT [28], Struck [57], TLD [25], DSST [4], FDSST [20], ASLA [58], SCT4 [37], KCF [3], CN [59], CT [60], L1APG [61], CSK [2], MIL [62], STC [63], IVT [64]. Among the 47 trackers, 8 same trackers (KCF, LCT, FDSST, SRDCF, Staple, MEEM, SAMF, CFNet) are reported for comparison of two different datasets. The total comparison results are shown in Figure 6. We also report comparison results with 26 non-deep-learning trackers in Figure 7 and several state-of-the-art deep learning-based trackers in Table 3.

Figure 6 shows that our proposed tracker achieves a 24.9% AUC score. Figure 7 illustrates that our tracker achieves the second accuracy among all listed state-of-the-art real-time trackers. What’s more, Table 3 shows some comparison results of our tracker and some state-of-the-art deep learning-based tracker. Our tracker runs at 30 FPS, while HCFT, MDNet, VITAL, and ECO all suffer from the high computational burden leading to no real-time speed. It is worth emphasizing that most deep learning-based trackers require expensive GPUs (e.g., RTX1080Ti) to train their models. However, not all researchers could afford this type of equipment, so our approach that uses an affordable CPU to run is still meaningful.

**Results on TrackingNet:** We evaluate our tracker on TrackingNet and report results on Table 4. The results show that our tracker achieves a 52.4% AUC score, which is comparable with some deep learning-based trackers (ECO 55.4% and CFNet 57.8%).

#### 3.3.2. Qualitative Evaluation

**Figure 8:**
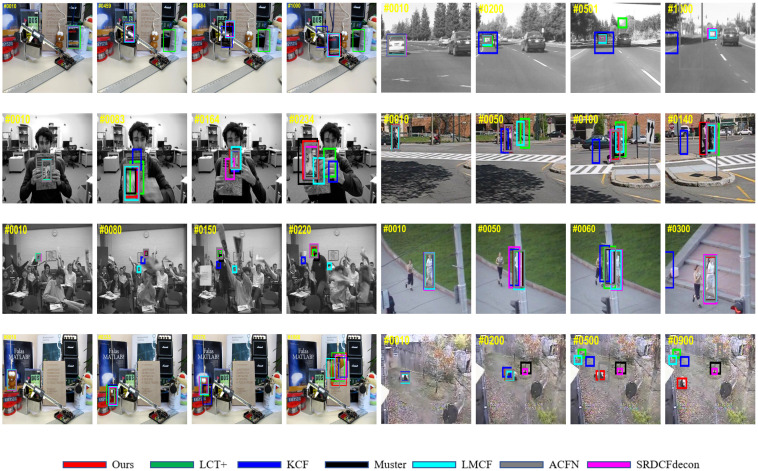
Tracking screenshoots of six state-of-the-art trackers and our tracking algorithm on 8 challenging sequences from OTB-2015 (from left to right and top to down are *Box*, *Car1*, *ClifBar*, *Couple*, *Freeman4*, *Jogging_2*, *Lemming* and *Panda*, respectively). The number in top left corner denotes frame index. Our algorithm is marked as red, we recommend checking this figure in color and amplification mode.

Figure 8 shows a qualitative comparison with the baseline KCF [3] and several state-of-the-art trackers SRDCF-decon [22], LMCF [29], LCT+ [34], ACFN [38], Muster [30] on eight challenging sequences. Among these eight videos, most main challenges present. The detail of challenges of these sequences are conducted in Table 2, and contain: scale variations (SV), occlusions (OCC), background clutter (BC), low resolution (LR), out-of-plane rotation (OPR), in-plane rotation (IPR), deformation (DEF) and out-of-view (OV). We also report the length of each sequence (L) in Table 5.

The baseline KCF only estimates the translation of the objective between the current frame and the previous frame yet keeps the scale all the same during the whole tracking process. Although KCF (marked as blue) has achieved some promising performances in normal situations such as the first 500 frames of *Car1* (Top right corner in Figure 8), yet finally loses its objective due to no scale adaption scheme. In contrast to KCF, our method (marked as red) presents a much favorable performance when encountering with huge scale variations in a particular sequence (*Car1*) as well as other challenging videos (*Couple*, *ClifBar*). Additionally, occlusions, one of the most difficult issues of visual tracking, always appear in the real-world, and presents a great challenge for all trackers. For example, seven of eight videos in this evaluation contains occlusions, particularly *Jogging_2*, *Box*, and *Lemming* where nearly the whole target are blocked in frames 50–58, 459–480 and 335–376, respectively. However, after the complete occlusion occurrence, our algorithm (LPCF) successfully differentiates the target from the clutter background, which can be seen in frames 60, 484, and 376, respectively. It deserves stressing that KCF updates the model frame-by-frame, inducing undesired inclusion of occlusions information. As a consequence, KCF loses its discriminative power and tracks the wrong target, occlusions instead.

The attentive reader may notice that ACFN (marked as gray) can distinguish the objective after occlusions without any decontamination approaches. ACFN, benefiting from its deep attentional network that selects a bunch of associated correlation filters, detects the target more frequently and robustly. Besides, other trackers listed in this evaluation all employe a scheme selecting training sample sets, which helps to improve their abilities against occlusions, background clutter, and other competitive attributes. Furthermore, SRDCFdecon achieves better overall performance (62.7% AUC score) than ours (61.0% AUC score), shown in Figure 4. The superiority of SRDCFdecon is demonstrated in most videos listed in Figure 8. This is because SRDCFdecon is developed based on SRDCF that integrates a spatial regularization component to alleviate the boundary effect. Although the spatial regularization scheme increases the discriminative power of the learned model but can not handle the out-of-plane rotation problem, shown in *ClifBar* 164th frame and *Panda* 200–900th frames. Compared to SRDCF and SRDCFdecon, our method LPCF utilizes the proposed tracking results validation scheme to reject those samples corrupting the discriminative power of our model more effectively and train our model using principal components of extracted samples that promotes the abilities against subtle disturbance, therefore successfully keeps tracking the objective (*Panda*).

In summary, our method performs favorably could be attributed to three reasons. (1) the tracking results validation scheme selects corrupted samples effectively; (2) the PCA strategy employed in our algorithm extracts the principal component of the training sample and significantly improves the abilities against subtle disturbance; (3) the scale estimation framework integrated with PCA strategy handles scale variations robustly.

## 4. Conclusions

In this paper, we offer a real-time, scale-adaptive, and robust tracker for visual tracking. To adapt to scale variations, we employ a generic scale estimation approach. Then we introduce a dimensionality reduction strategy to improve real-time performance and antagonize minor disturbance. Moreover, for the sake of preventing model drift induced by corrupted training samples, a locality preserving tracking validation method is proposed to ensure updating with proper training samples. Extensive experimental results on three large-sacle datasets demonstrate that the proposed decontamination method is effective, and the complete tracker performs favorably against some state-of-the-art methods and most existing decontamination trackers. Furthermore, it is worth emphasizing that the presented tracker not only performs favorably but also runs at a 30.63 FPS speed, which is sufficient for real-time applications.

## Figures and Tables

**Table 1:** Comaprison with basline trackers in distance precision (DP) at a threshold of 20 pixels, mean overlap precision (OP) at a threshold 0.5 of OPE evaluation, and mean frame-per-second (FPS) of 100 sequences. The best results and second highest values are highlighted by bold and underline.

Name	KCF [3]	FDSST [20]	KCF+FDSST	RKCF+FDSST	PKCF+FDSST	LPCF (Ours)
OP (%)	54.6	67.2	60.5	63.5	70.2	**75.3**
DP (pixel)	68.8	72.5	66.9	71.2	75.3	**78.9**
Speed (FPS)	**154.6**	52.7	53.7	44.5	59.4	30.3

**Table 2:** Comparison with baseline trackers on the test set of TrackingNet (511 sequences) in terms of precision, normalized precision and success (AUC). The best results and second highest values are highlighted by bold and underline.

Name	KCF	FDSST	KCF+FDSST	RKCF+FDSST	PKCF+FDSST	LPCF (Ours)
Precision (%)	42.14	42.64	40.09	41.32	42.14	**46.25**
Norm. Precision (%)	55.13	54.57	52.67	53.66	56.13	**58.80**
Success	45.24	49.69	47.69	48.45	50.67	**52.44**

**Table 3:** Comparison with deep learning-based trackers on the test set of LASOT (280 sequences) in terms of speed and success (AUC). The best results and second highest values are highlighted by bold and underline.

Name	LPCF (Ours)	Dsiam	MDNet	SiamRPN++	HCFT	ATOM	SiamMask	VITAL	ECO
Speed (FPS)	**30**	18	<1	21	10	**30**	22	<1	7
AUC (%)	24.9	33.3	39.7	49.5	25.0	**49.9**	46.7	39.0	32.4

**Table 4:** Comparison with state-of-the-art trackers on the test set of TrackingNet (511 sequences) in terms of precision, normalized precision and success (AUC). The best results and second highest values are highlighted by bold and underline.

Name	LPCF (Ours)	CFNet	MDNet	ECO	DaSiamRPN	SiamRPN++
Precision (%)	46.3	53.3	56.5	49.2	59.1	**69.4**
Norm. Precision (%)	58.8	65.4	70.5	61.8	73.3	**80.0**
Success	52.4	57.8	60.6	55.4	63.8	**73.3**

**Table 5:** Challenges of the experimental sequences.

Name	SV	OCC	BC	LR	OPR	DEF	IPR	OV	L
*Box*	*√*	*√*	*√*	*√*	-	*√*	*√*	*√*	1161
*Car1*	*√*	-	*√*	*√*	-	-	-	-	1020
*ClifBar*	*√*	*√*	*√*	-	-	-	*√*	*√*	472
*Couple*	*√*	*√*	-	*√*	*√*	-	-	-	140
*Freeman4*	*√*	*√*	-	-	*√*	-	*√*	-	283
*Jogging_2*	-	*√*	-	-	*√*	*√*	-	-	307
*Lemming*	*√*	*√*	-	-	*√*	-	-	*√*	1136
*Panda*	*√*	*√*	-	*√*	*√*	*√*	*√*	*√*	1000
